# In vitro activity of cefiderocol against Gram-negative aerobic bacilli in planktonic and biofilm form–alone and in combination with bacteriophages

**DOI:** 10.1038/s41598-025-01704-w

**Published:** 2025-05-16

**Authors:** Rima Fanaei Pirlar, Nexhmije Halili, Tina Travnik, Andrej Trampuz, Svetlana Karbysheva

**Affiliations:** 1 Center for Musculoskeletal Surgery, Charité—Universitätsmedizin Berlin, Corporate Member of Freie Universität Berlin and Humboldt-Universität zu Berlin, Berlin, Germany; 2 Berliner Hochschule für Technik (BHT), Berlin, Germany; 3 Biotechnical Faculty, University of Ljubljana, Ljubljana, Slovenia; 4 School of Medicine, Faculty of Health, Queensland University of Technology, Brisbane, QLD Australia; 5 Royal Brisbane and Women’s Hospital, Herston, QLD Australia

**Keywords:** Cefiderocol, Anti-biofilm activity, Phages, Multi-drug resistant pathogens, Microbiology, Medical research

## Abstract

Multi-drug resistant Gram-negative pathogens are increasingly difficult-to-treat perpetrators of infections. New, innovative, and more multifaceted therapies for the treatment of multi-drug resistant strains are thus urgent to hinder further drug resistance and mitigate deadly, untreatable infections. Our study aimed to investigate the efficacy of cefiderocol against Gram-negative aerobic bacteria alone and in combination with phages. The minimum inhibitory concentration (MIC) of cefiderocol was determined using the microdilution broth method, while the minimum biofilm bactericidal concentration was assessed using isothermal microcalorimetry. The combined effect of cefiderocol and phages was evaluated using colony-forming unit counts. Results demonstrated a notable antibacterial effect of cefiderocol, with 83.4% of tested strains exhibiting susceptibility. When combined with phages, the MIC of cefiderocol was reduced by 2–64-fold, indicating a synergistic interaction between the two agents. Furthermore, the combination therapy showed enhanced efficacy against biofilm compared to monotherapy with either cefiderocol or phages alone, leading to complete biofilm elimination in certain cases. This study highlights the potential of combining cefiderocol with phages as a strategy to combat multi-drug resistant Gram-negative bacterial infections. The observed synergy suggests that this combination therapy could improve treatment outcomes and help address the challenges of antibiotic resistance and biofilm-associated infections.

**Supplementary Information:**

The online version contains supplementary material available at 10.1038/s41598-025-01704-w.

## Introduction

Multi-drug resistant (MDR) Gram-negative pathogens represent a substantial challenge in the management of bacterial infections, particularly those associated with biofilm formation. These pathogens, including *Escherichia coli*, *Pseudomonas aeruginosa*, and *Acinetobacter baumannii*, exhibit various resistance mechanisms against a broad spectrum of antibiotics commonly employed in the treatment of Gram-negative bacterial infections, such as beta-lactams, carbapenems, and fluoroquinolones^1^. Biofilms exacerbate the difficulty of treating infections caused by MDR Gram-negative bacteria. The biofilm matrix not only shields the bacteria from immune responses but also limits the penetration of antibiotics, making the bacteria within biofilms up to 1,000 times more resistant to antimicrobial agents^2^. Addressing this issue requires urgent research into novel antimicrobial agents, alternative therapies, or strategies to disrupt biofilms and enhance antibiotic efficacy^3,4^.

Cefiderocol, a new generation cephalosporin, features a ferric-iron-binding siderophore catechol moiety, allowing it to act as a “Trojan horse” by using the iron transport system to enter Gram-negative bacteria, by passing porin channels. It is effective against MDR strains, including ESBL-producing and carbapenem-resistant *Enterobacterales*, and non-fermenting Gram-negative bacilli^5,6^. Cefiderocol offers a promising strategy for treating bacterial biofilm infections by disrupting biofilm formation through reducing ferric iron availability. As a siderophore antibiotic, cefiderocol targets iron, a vital nutrient for bacterial biofilm formation^7,8^. While cefiderocol exhibits strong bactericidal properties, it would be advantageous to develop a therapeutic strategy that preserves its efficacy while minimizing the selection pressure for resistance. Recent data from Germany show that cefiderocol resistance remains relatively uncommon. For example, a study conducted between 2019 and 2021 found that only 9 out of 108 carbapenem-resistant *P. aeruginosa* isolates were resistant to cefiderocol^9^. Another study from North Rhine–Westphalia looked at 115 difficult-to-treat Gram-negative isolates (collected from 2014 to 2021) and reported high cefiderocol susceptibility rates – over 80% in both *Enterobacterales* and non-fermenters^10^. 

Phage therapy for bactericidal treatment has recently been revived both in vitro and in vivo after decades of antibiotic use and consequent waves of antibiotic resistance^11,12^. For therapeutic investigations many studies use lytic phages as their lytic function is immediate. These phages cause bacterial lysis after every infection cycle without the capacity to integrate into the host genome^13^.

A combined multifaceted approach using the Trojan horse mechanism of cefiderocol and the lytic function of phages potentially offers a powerful and dynamic weapon against antibiotic and phage resistances^14^. A so-called phage-antibiotic synergy (PAS) has been observed in these types of combination therapies, whereby aside from reducing selective pressure for resistance, the two agents work synergistically, causing lower recorded minimum inhibitory concentration (MIC) of antibiotisc^15,16^. Moreover phages cause the degeneration of cell surface receptors responsible for the efflux of antibiotics, which in turn re-sensitizes the antibiotic^17^. Combination therapy is thus a possible solution for enhancing antimicrobial efficiency and reducing selection pressure^13,18^.

We hypothesize that cefiderocol might exhibit synergy with bacteriophages due to its unique mechanism of action as a siderophore cephalosporin. By actively utilizing iron transport systems to penetrate bacterial cells, cefiderocol may increase bacterial stress and alter membrane permeability, potentially enhancing phage adsorption and infection. It may lead to improved phage replication and lysis, deeper agent penetration into biofilms, and ultimately enhanced bacterial killing through a synergistic mechanism^13,19^.

In this study, we evaluated the antibacterial activity of cefiderocol in combination with lytic bacteriophages (or phages) against biofilm and planktonic strains of *E. coli*, *K. pneumoniae*,* P. aeruginosa*, and *A. baumannii*.

## Results

### MIC, MBC, and MBBC determination

The results of MIC and MBC determinations in planktonic bacteria are presented in Table 1. According to the MIC values obtained, cefiderocol susceptibility analysis revealed that all strains of *K. pneumoniae*,* P. aeruginosa*,* A. baumannii*, and one strain of *E. coli* (10 of the 12 tested strains) were susceptible to cefiderocol. Two strains of *E. coli* (2 and 3) fell into the intermediate category in accordance with CLSI criteria. The lowest and highest MIC and MBC values were observed for *P.aeruginosa* 2 (MIC = 0.06 µg/mL, MBC = 0.5 µg/mL) and *E. coli* 3 (MIC = 8 µg/mL, MBC = 64 µg/mL), respectively. The MBC/MIC ratio ranged from 4 (minimum) to 8 (maximum) across the *E. coli*, *K. pneumoniae*, and *P. aeruginosa* strains, whereas the highest MBC/MIC ratio of 16 was observed in *A. baumannii* (1 and 3).

**Table 1:** MIC and MBC values. Determined in planktonic bacterial strains by broth microdilution.

Strain	MIC (µg/mL)	MBC (µg/mL)	Interpretation
*E. coli* 1^a^	0.25	1	S
*E. coli* 2 ^a^	8	32	I
*E. coli* 3^b^	8	64	I
*K. pneumoniae* 1^a^	2	16	S
*K. pneumoniae* 2^a^	2	8	S
*K. pneumoniae* 3^a^	2	8	S
*P. aeruginosa* 1^a^	0.125	0.5	S
*P. aeruginosa* 2 ^a^	0.06	0.5	S
*P. aeruginosa* 3 ^a^	2	8	S
*A. baumannii* 1^a^	0.25	4	S
*A. baumannii* 2^a^	2	16	S
*A. baumannii* 3^a^	0.25	4	S

S, susceptible; I, intermediate; R, resistant.

^a^ Resistant to ureidopenicillins, third- or fourth-generation cephalosporins, carbapenems and fluoroquinolones; ^b^ Resistant to ureidopenicillins, third- or fourth-generation cephalosporins and carbapenems.

**Fig. 1:**
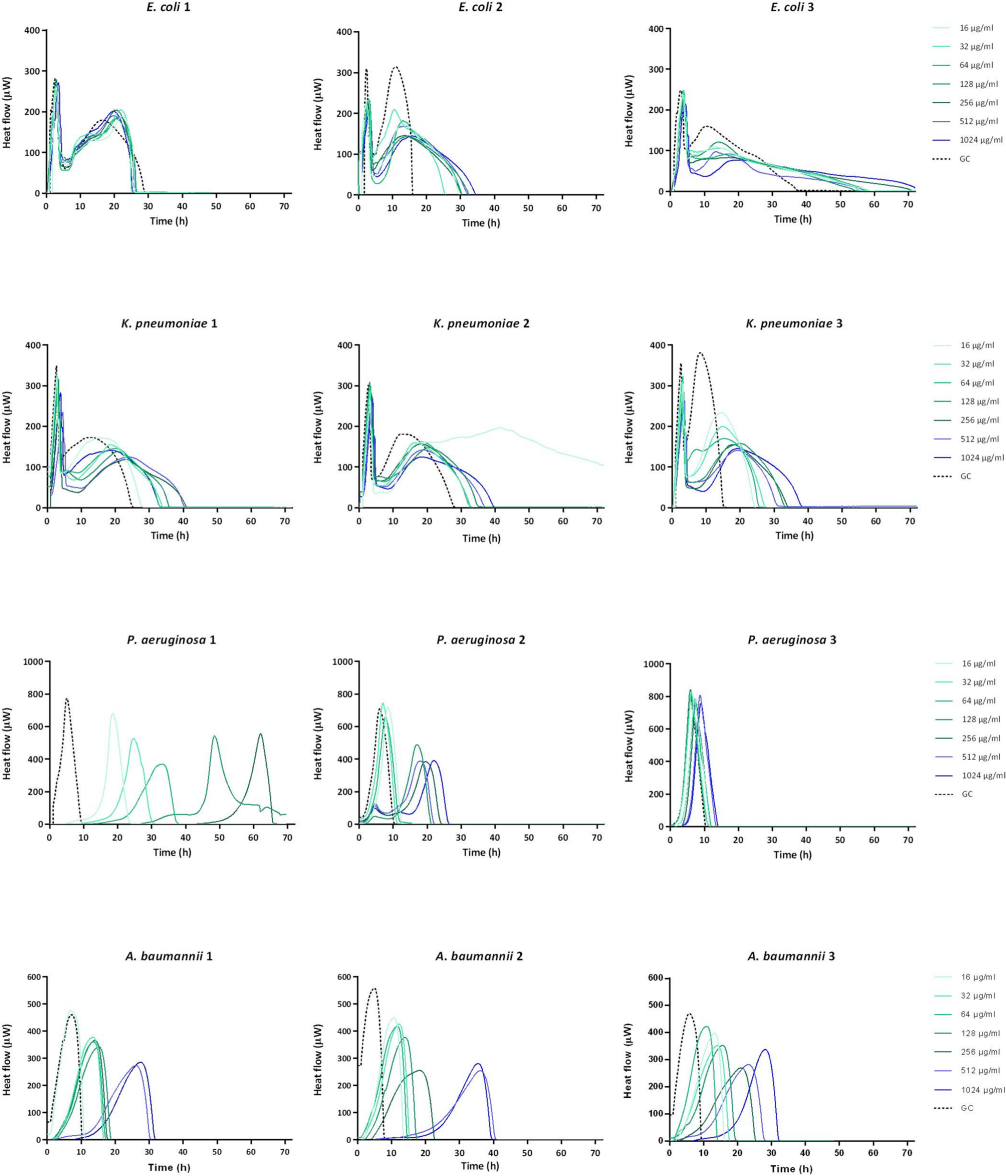
Calorimetric curves. Monitored heat flow (µW) of *E. coli* 1, 2, 3, *K. pneumoniae* 1, 2, 3, *P. aeruginosa* 1, 2, 3, and *A. baumannii* 1, 2, 3 biofilms treated with cefiderocol at different concentrations (16, 32, 64, 128, 256, 512, and 1024 µg/mL) as monotherapy. Each curve shows the heat flow produced over time by remaining viable bacteria in the biofilm after 24 h treatment. GC represents the growth control sample not exposed to any antimicrobials. Data of a representative experiment are reported.

Microcalorimetry anylysis showed that cefiderocol exhibited a dose-dependent activity against *P. aeruginosa* and *A. baumannii* biofilms, where increasing antibiotic concentrations correlated with increased antimicrobial activity. This was reflected by a delay in heat production or reduced heat flow compared to the untreated sample or absence of heat-flow at the highest concentrations (512 and 1024 µg/mL in *P. aeruginosa* 1). However, in *P. aeruginosa* 3, no significant antibiofilm effect was observed (Fig. 1). Although no significant anti-biofilm activity was observed against *E. coli* and *K. pneumoniae* biofilms, cefiderocol was able to reduce heat production in *E. coli* 2 by approximately 100 µW, even at the lowest concentration (16 µg/mL), compared to the growth control (Fig. 1).

The MBBC was > 1024 µg/mL for all strains except for *P. aeruginosa* 1 (512 µg/mL).

### Phage-cefiderocol synergistic effect against planktonic bacteria

**Fig. 2:**
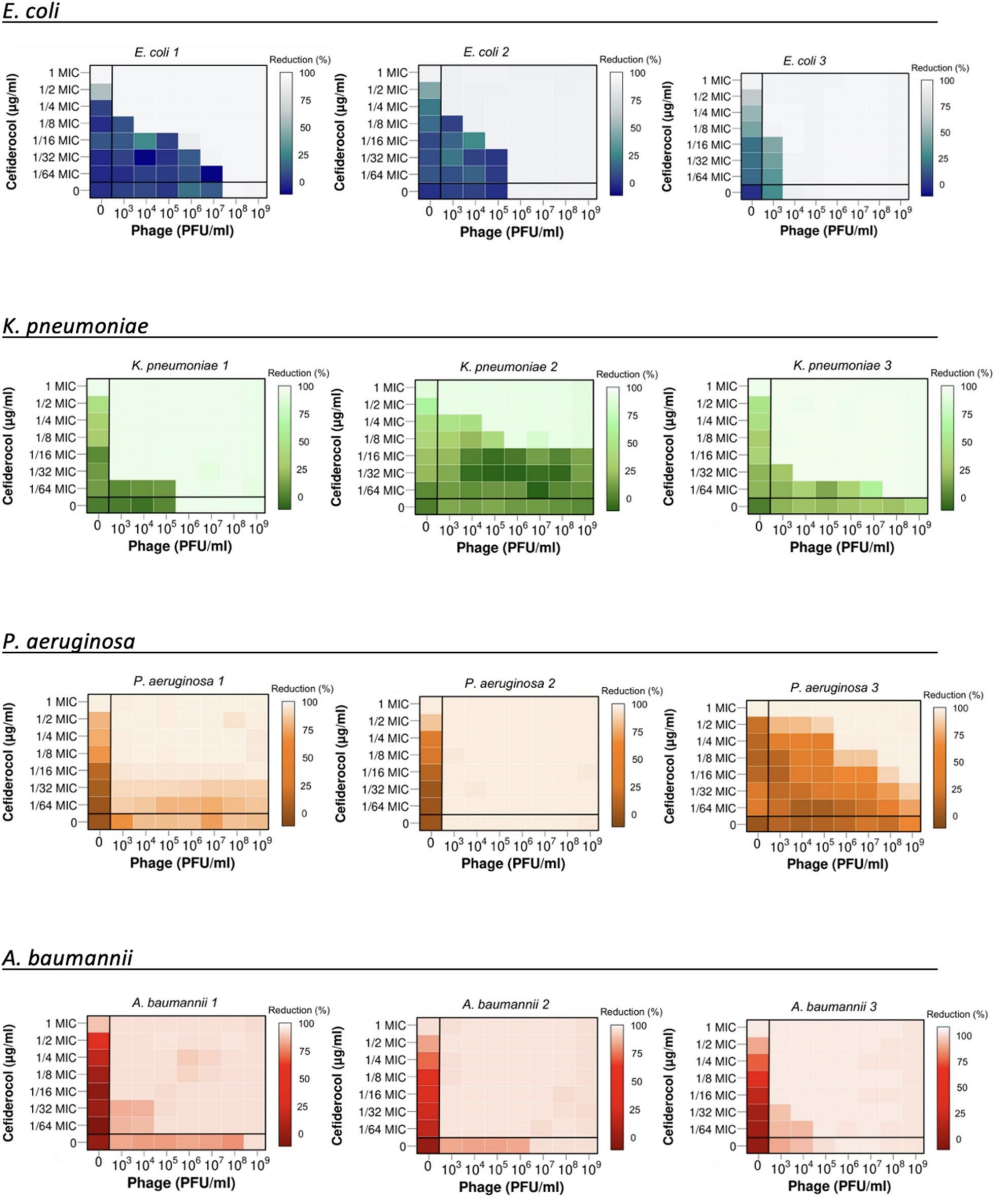
Phage-cefiderocol synergy. Synograms represent the mean reduction percentage of each treatment from three biological replicates: Reduction (%) = [(Odgrowthcontrol – ODtreatment)/ODgrowthcontrol] x 100. The regions between the solid lines represent the interacting regions of the phage and antibiotic, and the regions below the solid lines indicate phage and antibiotic alone.

In planktonic mode, different concentrations of cefiderocol (1/2 MIC to 1/64 MIC) were used alongside varying concentrations of the pahge against each tested strain (Fig. 2).

In *E. coli* 1, the combined application of cefiderocol with the minimum concentration (10^3^ PFU/mL) of the phage led to a 4-time reduction in the MIC (from 0.25 to 0.06 µg/mL). Both *E. coli* 2 and *E. coli* 3 exhibited intermediate susceptibility to cefiderocol. However, when cefiderocol was combined with even 10^3^ PFU/mL of the phages, its MIC values decreased by 4-fold (from 8 to 2 µg/mL) and 8-fold (from 8 to 1 µg/mL), respectively, shifting the MIC values into the susceptible range. For all three strains, the MIC decreased by more than 64-fold when the highest phage titer (10^9^ PFU/mL) was combined with cefiderocol.

Although the three *K. pneumoniae* strains exhibited the same susceptibility to cefiderocol, their responses to the phage-cefiderocol combination differed. The phage at 10^3^ PFU/mL reduced the MIC of cefiderocol for *K. pneumoniae* 1 by 32-fold (from 2 to 0.06 µg/mL), for *K. pneumoniae* 2 by 2-fold (from 2 to 1 µg/mL), and for *K. pneumoniae * 3 by 16-fold (from 2 to 0.125 µg/mL). At the phage titer of 10^9^ PFU/mL, the MIC was reduced by more than 64-fold in *K. pneumoniae* 1, 8-fold in *K. pneumoniae* 2, and 64-fold in *K. pneumoniae* 3.

The impact of phage on the reduction of cefiderocol MIC was particularly notable in *P. aeruginosa* 2, where even the lowest titer of the phage resulted in a more than 64-fold decrease in MIC (from 0.06 to < 0.0009 µg/mL). For *P. aeruginosa* 1, the maximum reduction observed was 16-fold (from 0.125 to 0.0075 µg/mL), regardless of whether the lowest or highest phage titer was used. Similarly, in *P. aeruginosa* 3, a 16-fold (from 2 to 0.125 µg/mL) reduction in MIC was achieved with the highest phage titer. However, in *P. aeruginosa* 3, the phage up to a titer of 10^6^ PFU/mL did not reduce the MIC when used in conjunction with cefiderocol.

For *A. baumannii* 1 and *A. baumannii* 3, similar outcomes were observed when cefiderociol was combined with the phage, resulting in 16-fold (from 0.25 to 0.015 µg/mL) MIC reduction, even when combined with a low phage titer of 10^3^ PFU/mL. The MIC of *A. baumannii* 2 was reduced by more than 64-fold across all tested phage titers.

### Phage-cefiderocol synergistic effect against biofilm bacteria

The anti-biofilm effect of cefiderocol and phage was assessed as a monotherapy or as a combination treatment.

**Fig. 3:**
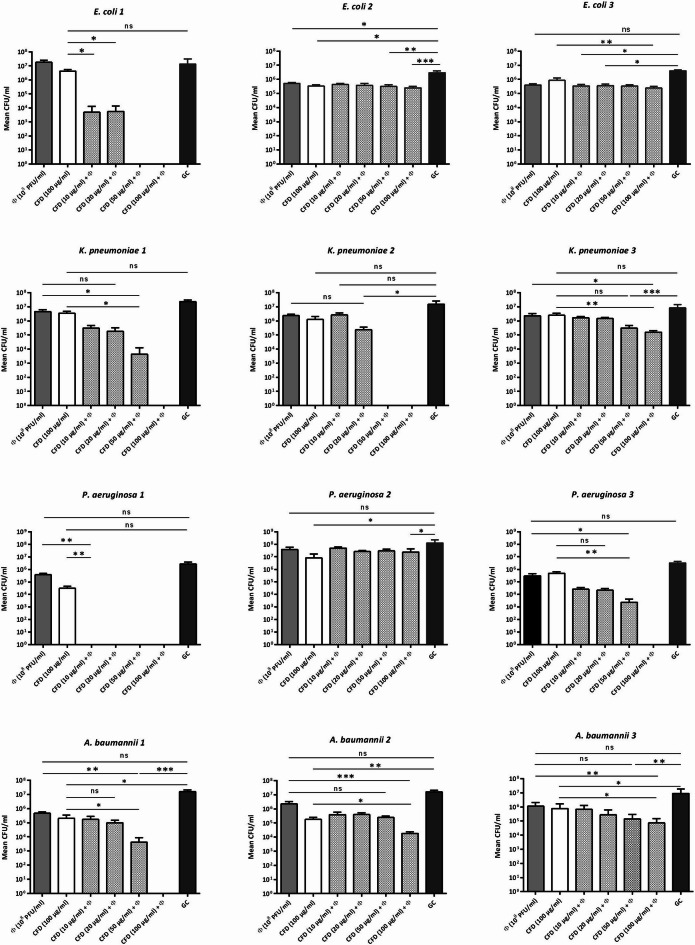
Anti-biofilm effect of cefiderocol-phage combination. Each column represents the CFU of remaining viable bacteria within the biofilm after 24 h of treatment with cefiderocol alone at 100 µg/mL, phage alone at 10^8^ PFU/mL, or cefiderocol-phage combinations. Error bars represent the standard deviation (SD) of the mean. CFD, cefiderocol; Φ, bacteriophage; GC, growth control. Data represent mean ± SD (*n* = 6). ***, *p* < 0.001; **, *p* < 0.01; *, *p* < 0.05.

The results showed that the combination of phage with low doses of cefiderocol below the MBBC (10, 20, 50, 100 µg/mL) revealed an inferior antimicrobial activity compared to the antibiotic or phage alone. A complete biofilm eradication was observed when cefiderocol was used at concentration of 10 µg/ml (for *P. aeruginosa* 1), 50 µg/mL (for *E. coli * 1 and *K. pneumoniae * 2), and 100 µg/mL (for *A. baumannii* 1, *K. pneumoniae* 1, and *P. aeruginosa * 3) in combination with phage. However, no complete eradication of the biofilm could be achieved with either the tested antibiotic or phage, even at the highest tested concentrations (Fig. 3).

The effects of cefiderocol and phage treatments varied across the different strains.

In *E. coli * 1, cefiderocol (100 µg/mL) alone did not significantly reduce CFU counts compared to the growth control. However, the combination of cefiderocol at a low dose (10 µg/mL) with phages significantly reduced CFUs (*p* < 0.05). While monotherapy with either cefiderocol or phages showed no significant reduction, higher cefiderocol doses (50 and 100 µg/mL) combined with phages completely eradicated the biofilm.

In *E. coli* 2 both cefiderocol and phage alone significantly reduced CFU counts compared to the growth control (*p* < 0.05), consistent with calorimetric data showing reduced heat production by cefiderocol. The combination of cefiderocol (50 and 100 µg/mL) with phage further reduced CFUs significantly compared to the control (*p <* 0.01 and *p <* 0.001, respectively), but not beyond the effect observed with cefiderocol or phage alone.

In *E. coli* 3, neither cefiderocol nor phage alone provide a significant anti-biofilm effect against the biofilm. However, the combination at even the lowest concentration (10 µg/mL) significantly reduced CFUs compared to the growth control, *p <* 0.05. Additionally, cefiderocol at 100 µg/mL combined with the phage significantly lowered CFUs compared to cefiderocol alone, *p <* 0.01.

In *K. pneumoniae* 1, neither cefiderocol nor phage alone produced a significant antibiofilm effect. However, the combination of cefiderocol at 50 µg/mL with phage significantly reduced CFUs compared to cefiderocol alone (*p <* 0.05). The combination therapy of cefiderocol at 100 µg/mL led to completely eradicated of the biofilm.

Similarly, in *K. pneumoniae* 2, neither cefiderocol nor phage alone showed significant antibiofilm activity. However, the combination of cefiderocol at 20 µg/mL with phage significantly reduced CFUs compared to the growth control (*p <* 0.05) and at concentrations of 50 and 100 µg/mL, the complete biofilm eradication was achieved.

In *K. pneumoniae* 3, cefiderocol alone did not result in significant CFU reduction, but the combination at 100 µg/mL with phage significantly reduced CFUs compared to cefiderocol alone, *p* < 0.01. However, no complete biofilm eradication was observed.

In *P. aeruginosa* 1, both cefiderocol and phage monotherapy significantly reduced CFUs compared to the growth control (*p* < 0.01), consistent with the calorimetric data from MBBC determination, and their combination at all tested cefiderocol concentrations completely eradicated the biofilm. In *P. aeruginosa* 2, cefiderocol alone significantly reduced CFUs (*p* < 0.05). However, combining it with phage did not enhance the antibiofilm effect. In *P. aeruginosa* 3, cefiderocol alone did not result in a significant reduction, which was expected based on the calorimetric data. However, the combination of cefiderocol at 50 µg/mL with phage significantly reduced CFUs (*p* < 0.01), and at 100 µg/mL completely eradicated the biofilm.

In *A. baumannii* 1, cefiderocol alone significantly reduced CFUs compared to the control (*p* < 0.05), while phage alone had no significant effect. The combination of cefiderocol at 50 µg/mL with phage significantly reduced CFUs compared to cefiderocol alone (*p* < 0.05), phage alone (*p* < 0.01), and the growth control (*p* < 0.001). At 100 µg/mL with phage, the combination completely eradicated the biofilm.

In *A. baumannii* 2, cefiderocol alone significantly reduced CFUs compared to the control (*p* < 0.01). However, the combination of cefiderocol at 100 µg/mL with phage enhanced the antibiofilm effect resulting in significantly reduction of CFUs, *p <* 0.05.

In *A. baumannii* 3, cefiderocol alone significantly reduced CFUs (*p* < 0.05).The combination of cefiderocol at 100 µg/mL with phage enhanced the antibiofilm effect leading to a significant reduction in CFU count compared to monotherapy of cefiderocol (*p* < 0.05) and phages (*p <* 0.01).

## Discussion

In this study, we explored the antimicrobial activity of cefiderocol against both planktonic and biofilm forms of four aerobic Gram-negative bacteria with 3 different strain each, namely *E. coli* (1, 2, and 3), *K. pneumoniae* (1, 2, and 3), *P. aeruginosa* (1, 2, and 3), and *A. baumannii* (1, 2, and 3). Additionally, we assessed the potential synergistic effect of combining cefiderocol with phages to target both planktonic and biofilm populations of these bacteria. This investigation aimed to elucidate how the interaction between cefiderocol and phages influences bacterial susceptibility and to determine whether this combination strategy could enhance antimicrobial efficacy, particularly against biofilm-associated infections.

In our study, all strains tested were MDR, yet they demonstrated susceptibility to cefiderocol at approximately 83%, while the remaining strains demonstrated intermediate susceptibility. Importantly, none of the tested strains were resistant to cefiderocol.

A MBC/MIC ratio of ≤ 4 observed in 50% of the strains, including *E. coli* 1 and 2, *K. pneumoniae* 2 and 3, and *P. aeruginosa* 1 and 3, indicates potent bactericidal activity of cefiderocol, which is consistent with findings from other studies^20,21^. In *E. coli* 3, *K. pneumoniae* 1, *P. aeruginosa* 2, and *A. baumannii* 2 strains with higher ratios (8 or 16), a higher concentration of cefiderocol is required to achieve bactericidal effects, though effective bacterial killing is still observed. *A. baumannii* 1 and 3 exhibited a MBC/MIC ratio of 16, suggesting the need for higher cefiderocol concentrations to achieve bactericidal outcomes. Overall, the relatively low MBC values confirm cefiderocol’s strong bactericidal potential particularly in our tested MDR difficult-to-treat Gram-negative pathogens. This finding aligns with previous research indicating the potent antibacterial efficacy of cefiderocol compared to other commonly used antibiotics in clinical settings^7^. While siderophore antibiotics like cefiderocol represent innovative therapeutic approaches against MDR Gram-negative bacilli^22^, the development of resistance mechanisms emphasizes the significance of using antibiotics sparingly^23^. Applying cefiderocol in combination with other antimicrobials might be a potential approach to mitigate the risk of resistance development and enhance treatment outcomes. Phages exhibit promising potential as an adjunctive therapy when combined with antibiotics, as evidenced by several compelling in vitro and in vivo studies^14,24–27^. These investigations have consistently demonstrated highly encouraging outcomes, further underscoring the potential synergy between phages and antibiotics in combating bacterial infections. When cefiderocol and phages are used together, they may have synergistic effects that speed up the removal of bacteria and reduce the emergence of resistant strains^28^. This is especially important for critically ill patients and those with few therapeutic alternatives^29,30^. Manohar et al. and Torres-Barceló et al. showed that phage-antibiotic combinations can control bacterial proliferation and antibiotic resistance by targeting different bacterial receptors^31,32^. They explained this by pointing out that when bacteria are invaded through various pathways, it is harder for them to become resistant^33,34^. Patricia J. Simner et al. indicated in a clinical case report with one patient infected with difficult-to-treat *P. aeruginosa* strain that phage augmented cefiderocol therapy. The patient received cefiderocol in conjunction with IV phage Pa14NPøPASA16, which resulted in sustained local site improvement and haemodynamic stability^24^.

It is noteworthy that our in vitro study represents the first investigation of the combination of phages with cefiderocol against four MDR Gram-negative bacteria in both planktonic and biofilm modes. This unique aspect of our research adds significant value to the existing body of literature, shedding light on the efficacy of this combination therapy across different bacterial growth states.

The combination of phage and cefiderocol showed promising results in their antimicrobial activity against MDR Gram-negative bacteria in planktonic and biofilm form. Our findings revealed synergistic interaction between the phage and cefiderocol. Overall, across all bacterial types, we consistently observed a reduction in the MIC of cefiderocol when combined with phages, underscoring a synergistic interaction between various phages across 12 distinct Gram-negative bacterial strains. Phages at the lowest titer used (10^3^ PFU/mL) exerted the effects with a 2–64 folds reduction in the MIC values almost for all the tested strains. Our findings clarified some important points: (i) the profound effect of phage diversity on cefiderocol activity, (ii) the general tendency of phage-mediated reduction in cefiderocol MIC, and (iii), the modulating role of host bacteria on phage-cefiderocol interactions. We propose that differences in burst size, a critical factor determining phage-mediated killing, may account for some of the observed variances in synograms among various phages. To clarify their combined effect on synergy, a thorough analysis of additional phage characteristics, such as adsorption rate and latent time, in addition to burst size, is necessary.

Furthermore, the anti-biofilm effect observed in this study exhibited diverse outcomes, ranging from negligible impact to complete elimination, despite the strains demonstrating susceptibility to cefiderocol and phages in planktonic mode. These findings align with prior research highlighting the intricate nature of biofilm structures, which can vary significantly due to differences in extracellular polymeric substance (EPS) composition among bacterial strains^35,36^. The varying responses observed underscore the complexity of biofilm-associated infections and emphasize the need for tailored treatment strategies that consider the unique characteristics of biofilm structures in different bacterial strains. This was particularly observed in *P. aeruginosa* 2, where combining phages with antibiotics had a strong effect against planktonic *P. aeruginosa* 2. The phage inhibited bacterial growth at a low concentration of 10³ PFU/mL, reducing the MIC of cefiderocol to over 64 µg/mL. However, the phage alone or in combination with cefiderocol did not significantly affect the biofilm formed by *P. aeruginosa* 2 except of the combination with the highest concentration of cefiderocol (100 µg/mL) and phages. In this strain cefiderocol showed minimal antibiofilm activity without any synergistic effect with phages, making it the least responsive strain among all tested. This point out the critical importance of investigating the antibiofilm effects of antibacterial agents, as the efficacy observed in planktonic form does not necessarily predict their effectiveness against the same strain in biofilm form.

PAS is influenced by multiple factors, including the antibiotic class and concentration, the bacterial host strain, and phage characteristics such as adsorption rate, latent period, and burst size. Interestingly, synergy observed with one antibiotic does not necessarily translate to another, even within the same mechanistic class, highlighting that molecular targets alone do not fully predict PAS outcomes. Similarly, phages with high genetic similarity (∼95%) can yield distinct synergistic effects, emphasizing the importance of specific phage-host interactions. The antibiotic concentration also plays a crucial role, as subinhibitory or varying doses can differentially modulate bacterial physiology and thus alter synergy levels. In cases of filamentation-associated PAS, increased bacterial surface area may enhance phage adsorption due to a higher density of accessible receptors. However, phages targeting different receptors on the same bacterial strain can still exhibit divergent PAS responses, suggesting that receptor specificity and availability during filamentation are also critical factors^14,37^. Despite this, detailed studies on receptor density changes under filamentation conditions remain limited.

Previous studies on PAS have shown that certain β-lactam antibiotics, including cephalosporins such as ceftazidime, can enhance phage activity by inducing bacterial filamentation and altering cell wall synthesis^16,38,39^. This morphological transformation, often triggered by β-lactams binding to penicillin-binding proteins (PBPs) – especially PBP3, which is critical for septum formation during bacterial division – leads to elongated, filamentous cells. Such filamentation has been associated with increased phage adsorption and replication, likely due to the larger bacterial surface area and altered receptor presentation. Cefiderocol, a siderophore-conjugated cephalosporin, shares a similar mechanism, targeting PBP3 and interfering with peptidoglycan synthesis, thereby contributing to bacterial cell death during division^13,40–43^. Moreover, cephalosporins have been demonstrated to enhance phage particle production, potentially by creating favorable conditions for phage replication. The synergistic interaction may also stem from phage-derived enzymes disrupting membrane integrity and cefiderocol’s unique iron uptake-based entry mechanism could jointly weaken bacterial defenses and biofilm structure. This multifactorial interplay likely facilitates deeper agent penetration and increases bacterial susceptibility, thereby enhancing the overall antimicrobial efficacy of the combination treatment.

Our results highlighted that bacterial strain and its growth mode, phage type and density affected the treatment outcome. Therefore, the development of personalized therapies is essential to ensure therapeutic efficacy in patients^44^. However, as it was shown in our study, the selection of appropriate phage-antibiotic combinations presents an additional challenge, as these combinations do not consistently exhibit synergistic effects across all bacterial strains. Further comprehensive in vivo and in vitro studies involving a wider range of bacterial species are required to facilitate the development of standardized therapeutic formulations^13^.

Taken together, the treatment of MDR infections remains a major challenge for clinicians and an ongoing threat to public health. These infections might benefit from being treated with an optimized combination therapy^13^. The combination of cefiderocol, a new antibiotic with low resistance rates and a unique mechanism of action, along with phages, a novel antimicrobial agent, holds promise for effectively combating Gram-negative bacterial infections, particularly biofilm-associated infections. When used together, cefiderocol and phages may offer a synergistic approach to tackling bacterial infections, including those caused by biofilms, where traditional antibiotics often struggle to reach and eliminate bacteria effectively. Further research into this combination therapy could pave the way for innovative and tailored treatments against challenging bacterial infections.

Two main limitations to our study should be considered. Firstly, the isolated phages were not fully characterized and sequenced to determine their genotypes. This analysis would have provided valuable insights into the diversity and similarities among the phages used in our study. However, the detailed characterisation was not within the scope of this research as the focus of our study was to evaluate the combination therapy of cefiderocol and phages against MDR Gram-negative bacteria. Secondly, only cefiderocol susceptible bacterial strains were tested. To better assess the effectiveness of this treatment approach, future studies should include testing against bacterial strains that are known to be resistant to either cefiderocol or phages. Evaluating the efficacy of the combination therapy against resistant strains would provide a more comprehensive understanding of its potential applications and limitations in clinical settings.

In conclusion, cefiderocol alone shows good bactericidal activity against tested MDR planktonic Gram-negative bacteria. Conversely, the application of monotherapy with cefiderocol or phages does not exhibit biofilm elimination effect. The conjunction of cefiderocol with phages demonstrates a heightened efficacy against planktonic and biofilm bacteria, resulting in the significant cefiderocol MIC reduction and complete elimination of the biofilm in certain cases, respectively. Cefiderocol conjunction with phages exhibits superior antibacterial and anti-biofilm activity, showing the potentially promising therapy that might be incorporated in clinical practice and improve the outcome of MDR bacterial infection treatment.

## Materials and methods

### Bacterial strains, bacteriophages, and antibiotic

This study involved 12 MDR clinical isolates including three isolates each of *E. coli*, *K. pneumoniae*,* P. aeruginosa*, and *A. baumannii*, provided by Labor Berlin–Charité Vivantes GmbH, Berlin, Germany. Eleven of twelve strains exhibit resistance to four antibiotic groups including ureidopenicillins, third- or fourth-generation cephalosporins, carbapenems, and fluoroquinolones (4MDRO, multidrug-resistant organism) and one strain, *E. coli* 3, showed resistance to three antibiotic groups including ureidopenicillins, third- or fourth-generation cephalosporins, and carbapenems (3MDRO)^45^. These MDR clinical isolates were selected based on their real-world resistance profiles (3MDRO/4MDRO) to reflect current clinical challenges rather than using standardized repository strains.

Cefiderocol powder, obtained from Shionogi, Osaka, Japan, was reconstituted in sterile 0.9% saline (Merck KGaA in Darmstadt, Germany), immediately prior to utilization. The bacterial strains were preserved at -80 °C using a cryovial bead preservation system (Microbank; Pro-Lab Diagnostics, Canada). Phages specific to the bacterial strains were isolated from sewage at a wastewater treatment plant and subsequently employed in the experimental procedures. Against each bacterium, one phage was isolated, meaning a total of 12 phages were used in the study, as we had 12 bacterial strains.

The phage isolation procedure employed an enrichment method detailed in a previous study^46^. To ensure phage purity, four consecutive cycles of single-plaque isolation were conducted on the bacterial host strain. Subsequently, the phage was cultured in a liquid medium, with bacterial strains grown in tryptic soy broth (TSB) (US Biological, Eching, Germany) overnight at 37 °C. A single phage plaque selected from a plate lysate was resuspended in 1 mL of filter-sterilized SM buffer (10 mM Tris-HCl, pH 7.8 (Carl Roth GmbH, Karlsruhe, Germany); 1 mM MgSO4 (Merck KGaA, Darmstadt, Germany)), followed by incubation at 4 °C for 1 h.

In the next step, 0.2 mL of an overnight bacterial culture was inoculated into 20 mL sterile TSB and incubated with agitation at 37 °C until reaching an OD_600_ of 0.4. Then, 0.1 mL of SM buffer containing the phage plaque was added, and the culture was incubated at 37 °C with agitation for approximately 5 h or until the culture clarified. The resulting phage lysate was centrifuged at 4000× g for 20 min, and the supernatant was filter-sterilized using a 0.22 μm filter. Phage precipitation was achieved by adding 8% w/v polyethylene glycol (PEG-8000; Pan-Reac AppliChem, Darmstadt, Germany) at 4 °C overnight, followed by centrifugation at 13,000× g at 4 °C for 40 min using a fixed-angle rotor Eppendorf 5810 R centrifuge (Eppendorf, Hamburg, Germany). After centrifugation, the pellet was resuspended in SM buffer, filtered, and stored at 4 °C until further use. Titration was conducted on the host strain to determine the corresponding phage titer.

All specific isolated phages consistently formed clear plaques in repeated assays, with no turbid zones or spontaneous bacterial regrowth, features commonly associated with lytic phages^47,48^. Additionally, the high phage titers observed in our study (10⁹ − 10¹³ PFU/mL) further support the assumption of a lytic life cycle.

We selected TSB for bacterial growth and phage production because it is a nutrient-rich, non-selective medium recommended by ATCC (American Type Culture Collection) for cultivating diverse Gram-negative clinical isolates. Moreover, previous studies have demonstrated that such media enhance phage yield and improve plaque morphology^49^.

### MIC, MBC, and MBBC determination

The micro-dilution broth method (performed using iron-depleted cation-adjusted Mueller-Hinton broth) by subculturing to agar plates was employed to determine the minimum inhibitory concentration (MIC) and minimum bactericidal concentration (MBC) of cefiderocol^16^. The MIC methology and susceptibility interpretation were determined according to the Clinical and Laboratory Standards Institute (CLSI)^50^. Consequently, the tested bacterial strain was considered susceptible to cefiderocol when MIC ≤ 4 µg/mL. Standard strains including *E. coli* ATCC 25922, *K. pneumoniae* ATCC 13883, *P. aeruginosa* ATCC 27853, and *A. baumannii* ATCC 13304 were used for quality control.

Isothermal microcalorimetry was used to determine the antimicrobial activity of cefiderocol against the biofilm, as previously reported^51^. The heat monitored in this assay is related to the metabolic activity of surviving cells within biofilm incubated in a fresh medium. Bacterial biofilms were formed on sterile 1.5 mm sintered porous glass beads (ROBU, Hattert, Germany) by placing each bead in a single well of a 24-well plate (Corning Inc., Corning, NY, USA) containing 1 mL TSB inoculated with 1:100 dilution of bacteria and incubated at 37 °C and 150 rpm for 24 h. Subsequently, glass beads were washed three times with sterile 0.9% saline to remove non-adherent planktonic cells before being transferred to 24-well plates containing 1 mL of iron-depleted Muller Hinton broth (ID-MHB) with increasing concentrations of cefiderocol (16, 32, 64, 128, 256, 512, 1024 µg/mL). Samples were incubated at 37 °C for 24 h. After treatment, beads were gently washed three times with 0.9% sterile saline to remove any presence of antimicrobial agent and placed in 1 mL of fresh TSB in air-tight sealed ampoules. Heat flow (µW) was continuously measured at 37 °C for up to 72 h by isothermal microcalorimetry.

The minimum biofilm bactericidal concentration (MBBC) is defined as the lowest antimicrobial concentration that strongly reduced the number of viable bacterial cells within the biofilm, therefore leading to undetectable heat flow values within 72 h monitoring.

### Phage-cefiderocol combinations against planktonic bacterial cells

To evaluate the synergistic effect, a matrix-based combination assay was performed. Cefiderocol was prepared at decreasing sub-MIC concentrations (ranging from 1/2 MIC to 1/64 MIC), and the phage concentrations ranged from 10³ to 10⁹ PFU/mL. The experimental setup involved introducing 50 µl of phages and 50 µl of cefiderocol into each well, followed by the addition of 100 µl of a bacterial suspension at a density of 10^6^ CFU/mL. The plates were then incubated overnight at 37 °C, and absorbance was measured after 24 h. To construct synograms, absorbance readings from three biological replicates were normalized against the negative control. The readings from treated wells were then subtracted from those of the positive control (no treatment) to calculate percent reduction using the formula: Reduction (%) = [(ODgrowthcontrol - ODtreatment)/ODgrowthcontrol] x 100^14^. The resulting numeric values were processed and visualized as synograms (heat maps) using R Studio (version 2022). To facilitate a clearer interpretation of the synergistic interactions, we categorized the results based on the calculated percent reduction and used corresponding color codes to visually represent the synergy. The corresponding numeric data used to generate these synograms are provided as supplementary material (Supplementary Data S1).

#### Phage-Cefiderocol combinations against bacterial biofilm

For each bacterial strain the 24 h-old-biofilms were formed on porous glass beads as previously described^52^. Biofilms were exposed to either phage (10^8^ PFU/mL) or cefiderocol at concentration of 100 µg/mL as monotherapy. For phage-antibiotic combinations, biofilms were exposed to a simultaneous co-incubation with phage (10^8^ PFU/mL) and cefiderocol at various concentrations below the MBBC (10, 20, 50, and 100 µg/mL) prepared in fresh ID-MHB in a final volume of 1 mL and incubated at 37 °C for 24 h. After an overall incubation of 24 h, treated biofilm beads were rinsed three times with 0.9% saline, and further investigated for quantification of the remaining bacteria by sonication^53^. The number of bacteria cells adhering to the glass beads was determined by transferring washed beads to Eppendorf tubes filled with 0.5 mL PBS, followed by 1 min sonication in an ultrasound bath at 40 kHz and 0.2 W/cm^2^ (BactoSonic, BANDELIN electronic, Berlin, Germany). Ten-fold serial dilutions were plated on TSA and colonies were counted after 18 to 24 h incubation at 37 °C. Two biological replicates with technical triplicates were carried out.

Representative images of biofilms were not included in this study; however, we prioritized CFU enumeration for its quantitative accuracy and reproducibility.

We selected a cefiderocol concentration range of 10–100 µg/mL for our antibiofilm experiments to ensure clinical relevance and to reflect drug levels achievable in human serum. A 2 g infusion of cefiderocol leads to a maximum serum concentration (C_max_) of 89.7–156 µg/mL, depending on the infusion time. Considering that about 40% of cefiderocol binds to proteins, the free active concentration in the body would be around 54 to 94 µg/mL^53–55^. Thus, our in vitro exposure concentrations (10–100 µg/mL) remain within the range of C_max_ observed in humans and do not exceed the reported average C_max_ .

### Statistical analyse

Quantitative data were presented as mean ± standard deviation (SD) for each strain and group separately and plotted as bacterial count (CFU/mL). The normality of data distribution was examined by Shapiro-Wilk test. The ANOVA test was performed to compare the examined groups and in case of non-normal distribution of the variables, the Kruskal Wallis test was performed. The statistical analysis and graphs creation were carried out by IBM SPSS Ver. 21 and GraphPad Prism (version 8; GraphPad, La Jolla, CA, USA). The significance level was considered as *p* < 0.05.

## Electronic supplementary material

Below is the link to the electronic supplementary material.

Supplementary Material 1

## Data Availability

The datasets used and/or analysed during the current study are available from the corresponding author on reasonable request. All data generated or analysed during this study are included in this published article.
