# Supplementary material for: In vitro activity of cefiderocol against Gram-negative aerobic bacilli in planktonic and biofilm form–alone and in combination with bacteriophages
Source: Sci Rep. 2025 May 16;15:17105. doi: 10.1038/s41598-025-01704-w (PMC12084636; doi:10.1038/s41598-025-01704-w)

In vitro activity of cefiderocol against Gram-negative aerobic bacilli in planktonic and biofilm form–alone and in combination with bacteriophages

Rima Fanaei Pirlar[1], Nexhmije Halili[2], Tina Travnik[3], Andrej Trampuz[1,4,5], Svetlana Karbysheva[1*]

In the synogram numerical tables, green-colored wells indicate either additive or synergistic interactions, defined as a >10% reduction compared to the most effective monotherapy at the respective concentration—whether phage or cefiderocol. Blue wells represent wells where the bacterial inhibition was comparable to that observed with the strongest monotherapy alone, suggesting that the use of a second agent does not provide added benefit; therefore, such wells were not classified as synergistic or additive. Pink wells denote an indifferent effect, where the combination therapy neither enhanced nor impaired bacterial inhibition compared to individual treatments. Rare instances of antagonism, where the combination performed worse than the monotherapies, were also included in this category due to their negligible occurrence.

Reduction (%)								
MIC	88	94	94	95	95	94	95	94
1/2 MIC	45	94	94	95	95	94	95	94
1/4 MIC	17	92	94	94	90	92	95	95
1/8 MIC	-3	94	94	94	91	94	95	95
1/16 MIC	-3	94	94	93	95	95	95	94
1/32 MIC	1	83	84	93	94	95	95	94
1/64 MIC	-24	85	86	94	94	95	95	94
GC	0	82	81	80	82	81	79	94
		10Λ3	10Λ4	10Λ5	10Λ6	10Λ7	10Λ8	10Λ9

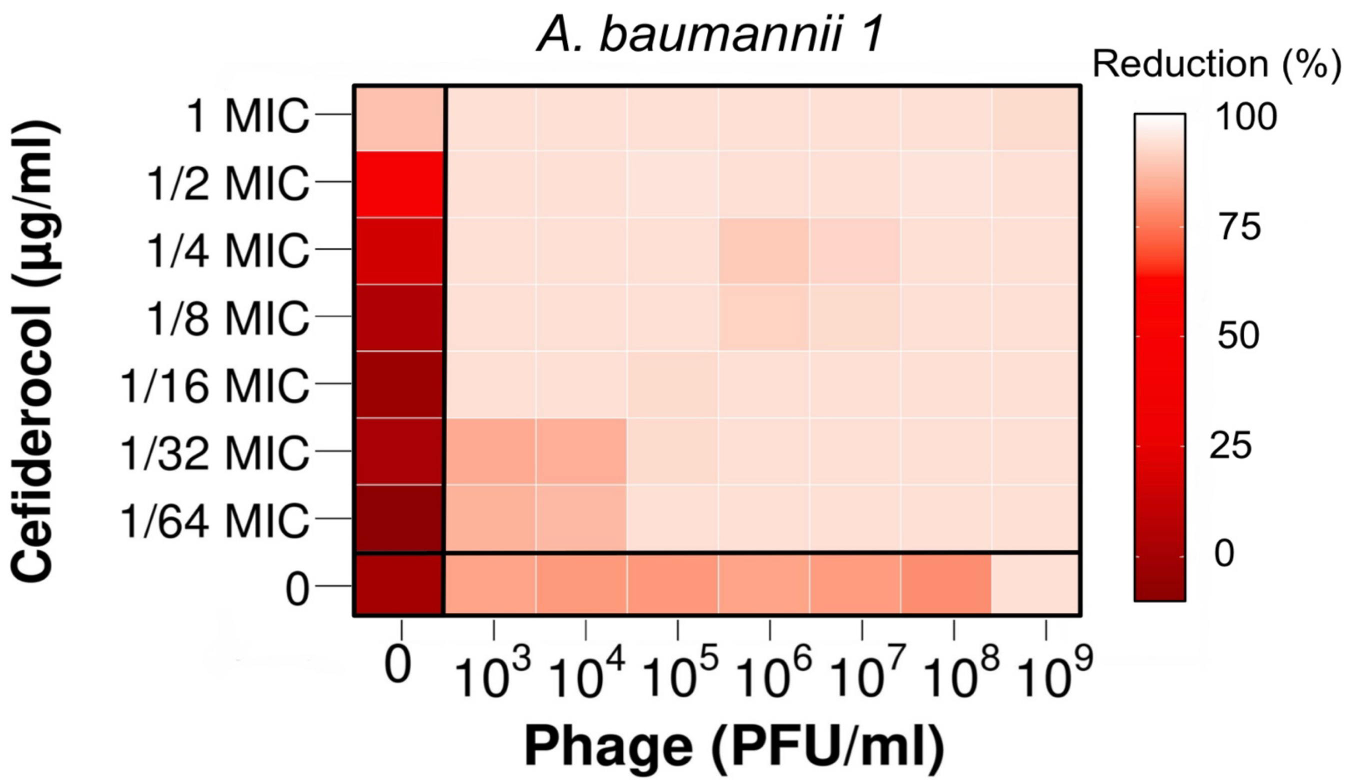

Reduction (%)								
MIC	95	95	95	95	95	95	95	94
1/2 MIC	82	95	95	95	95	95	95	94
1/4 MIC	71	95	95	95	95	95	95	94
1/8 MIC	42	95	95	95	95	95	95	94
1/16 MIC	33	95	95	95	95	95	93	94
1/32 MIC	23	95	95	95	95	95	94	93
1/64 MIC	17	95	95	95	95	93	95	94
GC	0	83	82	82	83	94	94	94
		10^3	10^4	10^5	10^6	10^7	10^8	10^9

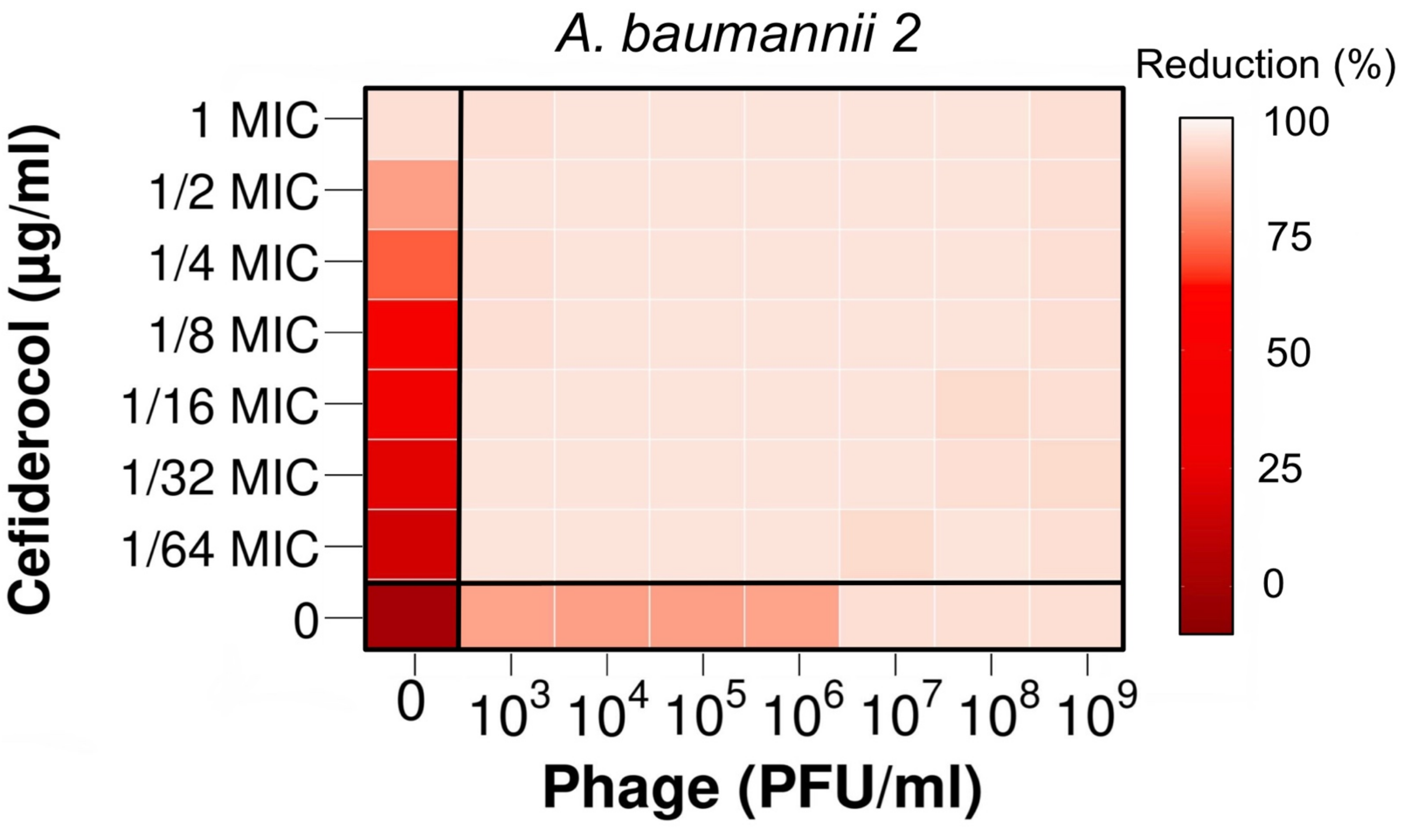

**Reduction** (%)								
MIC	96	96	96	96	96	96	96	95
1/2 MIC	83	96	96	96	96	96	95	95
1/4 MIC	69	96	96	96	96	95	95	95
1/8 MIC	37	96	96	96	96	96	96	95
1/16 MIC	16	96	96	96	96	95	95	95
1/32 MIC	6	88	96	96	96	96	96	95
1/64 MIC	4	86	86	96	95	96	95	95
GC	0	83	86	94	96	96	95	95
		10Λ3	10Λ4	10Λ5	10Λ6	10Λ7	10Λ8	10Λ9

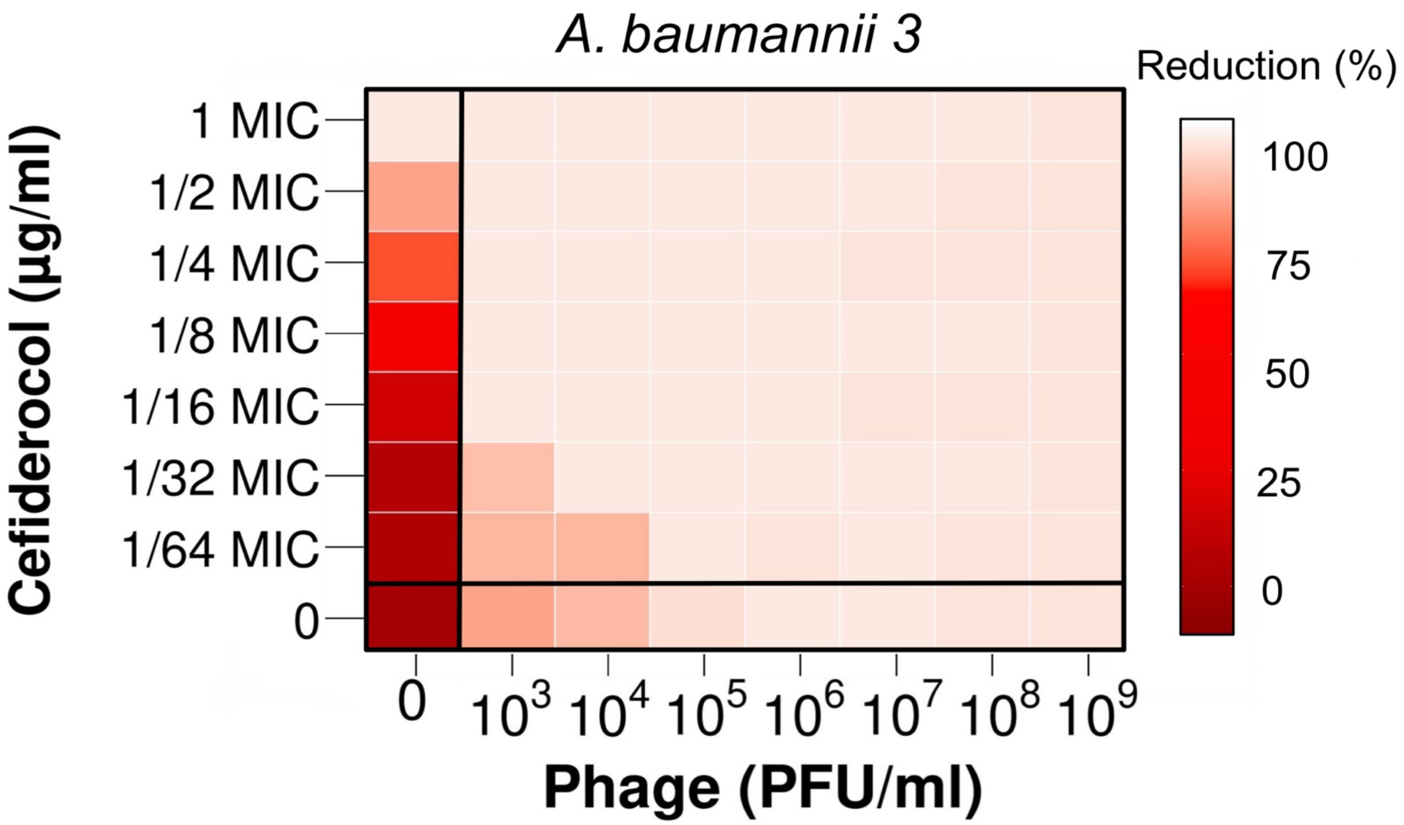

**Reduction** (%)								
MIC	94	94	94	94	94	94	94	94
1/2 MIC	58	94	94	94	94	94	94	94
1/4 MIC	6	94	94	94	94	94	94	94
1/8 MIC	-1	10	94	94	94	94	94	94
1/16 MIC	11	12	28	6	88	94	94	94
1/32 MIC	-2	6	-9	1	13	93	94	94
1/64 MIC	-2	-1	5	4	9	-9	94	94
GC	0	5	7	2	20	13	94	93
		10Λ3	10Λ4	10Λ5	10Λ6	10Λ7	10Λ8	10Λ9

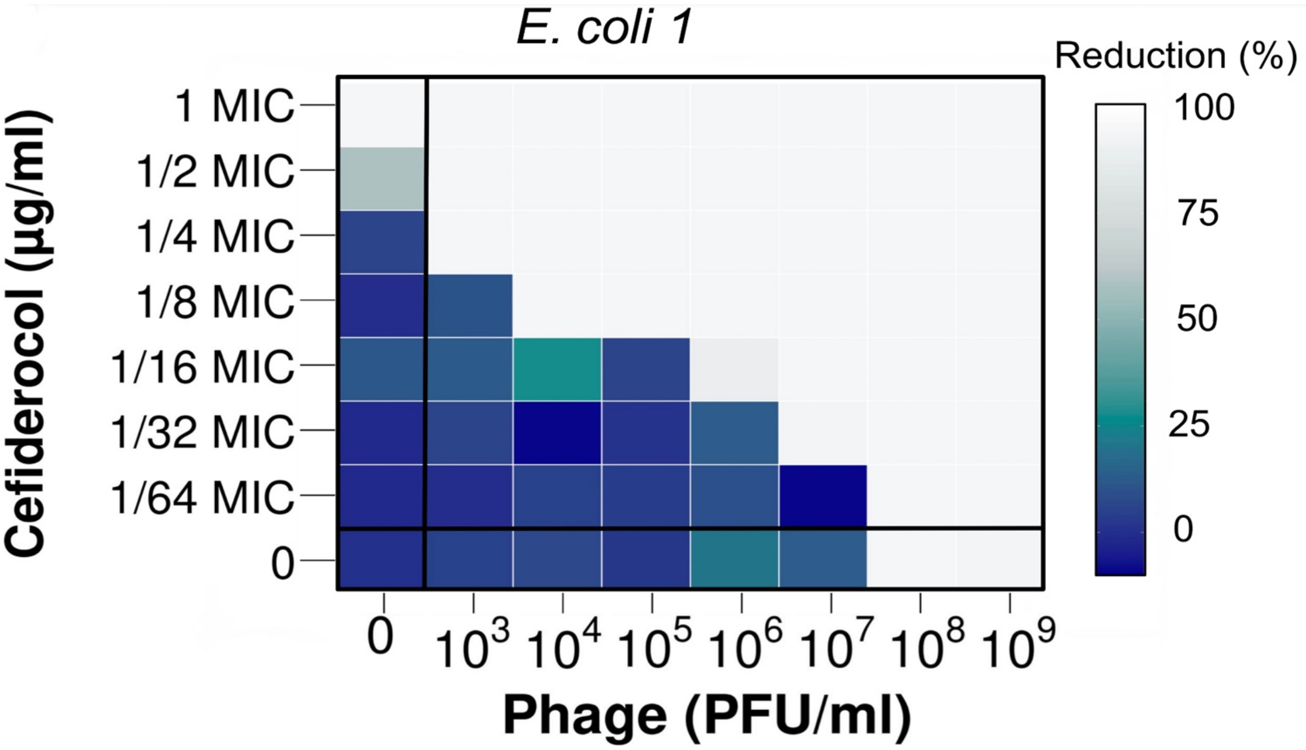

**Reduction** (%)								
MIC	91	90	91	91	90	90	90	90
1/2 MIC	45	90	90	90	91	91	90	91
1/4 MIC	22	90	90	91	91	91	91	91
1/8 MIC	18	6	90	91	91	91	91	91
1/16 MIC	7	16	28	91	91	91	91	91
1/32 MIC	9	21	6	2	91	91	91	91
1/64 MIC	11	10	15	4	91	91	91	91
GC	0	5	6	0	91	91	91	91
		10^3	10^4	10^5	10^6	10^7	10^8	10^9

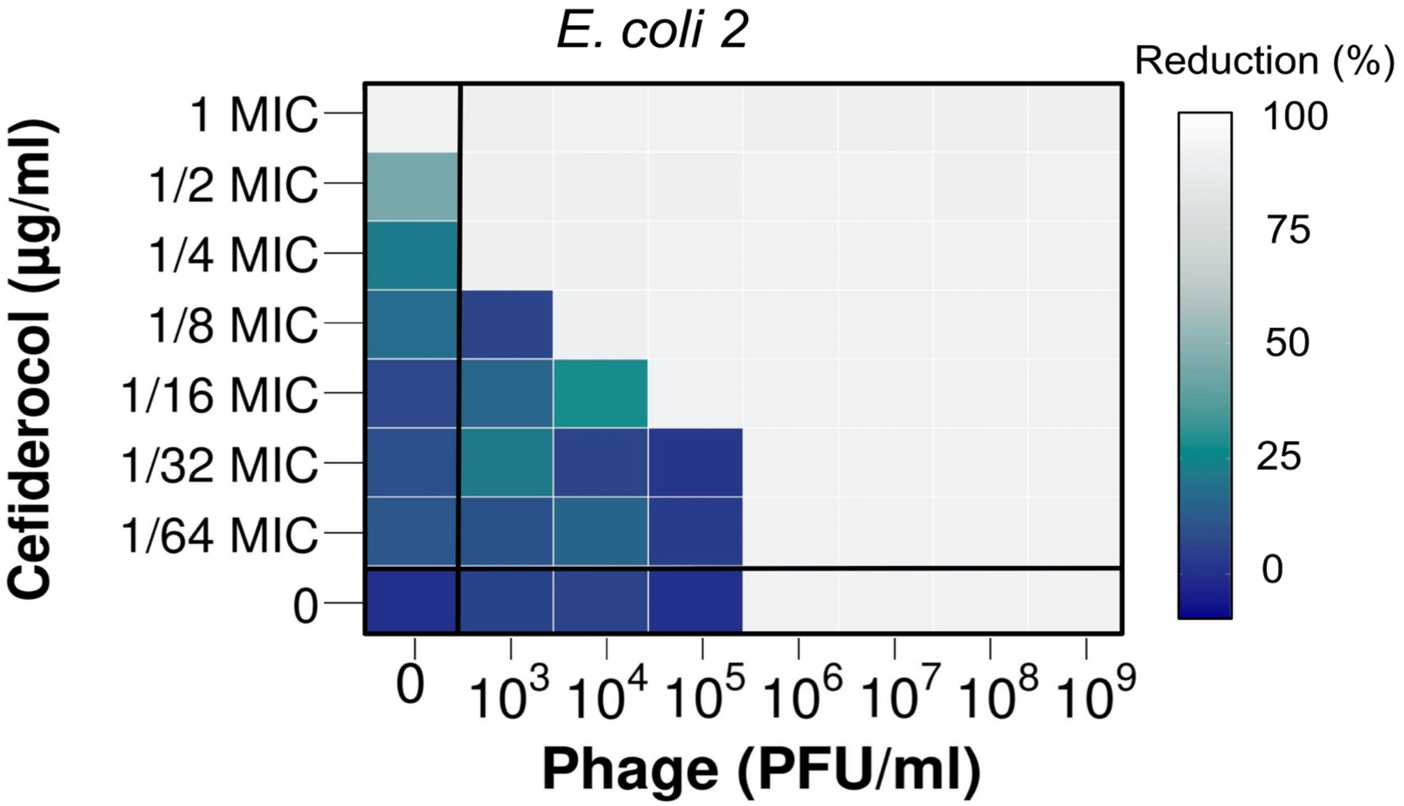

Reduction (%)

MIC	93	93	93	92	93	94	94	93
1/2 MIC	63	93	93	92	94	94	94	93
1/4 MIC	51	94	94	92	94	94	94	93
1/8 MIC	42	93	94	92	94	94	94	93
1/16 MIC	20	44	94	92	94	94	94	93
1/32 MIC	20	40	92	92	94	94	94	93
1/64 MIC	17	34	92	92	94	94	94	93
GC	0	29	90	92	94	94	93	93
		10^3	10^4	10^5	10^6	10^7	10^8	10^9

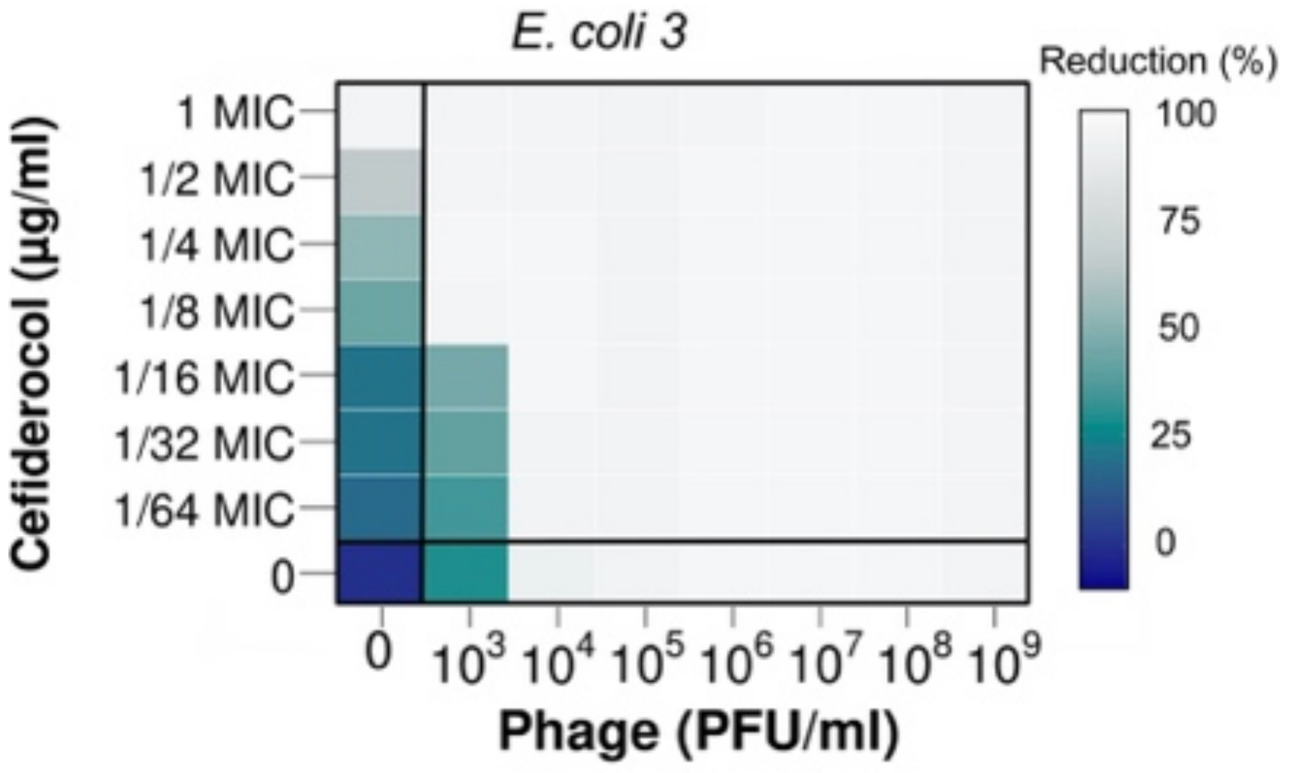

Reduction (%)								
MIC	92	92	92	92	92	92	92	91
1/2 MIC	45	92	92	92	92	92	92	91
1/4 MIC	32	92	92	92	92	92	92	91
1/8 MIC	29	92	92	92	92	92	92	91
1/16 MIC	5	92	92	92	92	91	92	91
1/32 MIC	10	92	92	92	92	89	92	91
1/64 MIC	11	5	8	10	92	91	92	89
GC	0	0	-4	4	92	92	92	91
		10Λ3	10Λ4	10Λ5	10Λ6	10Λ7	10Λ8	10Λ9

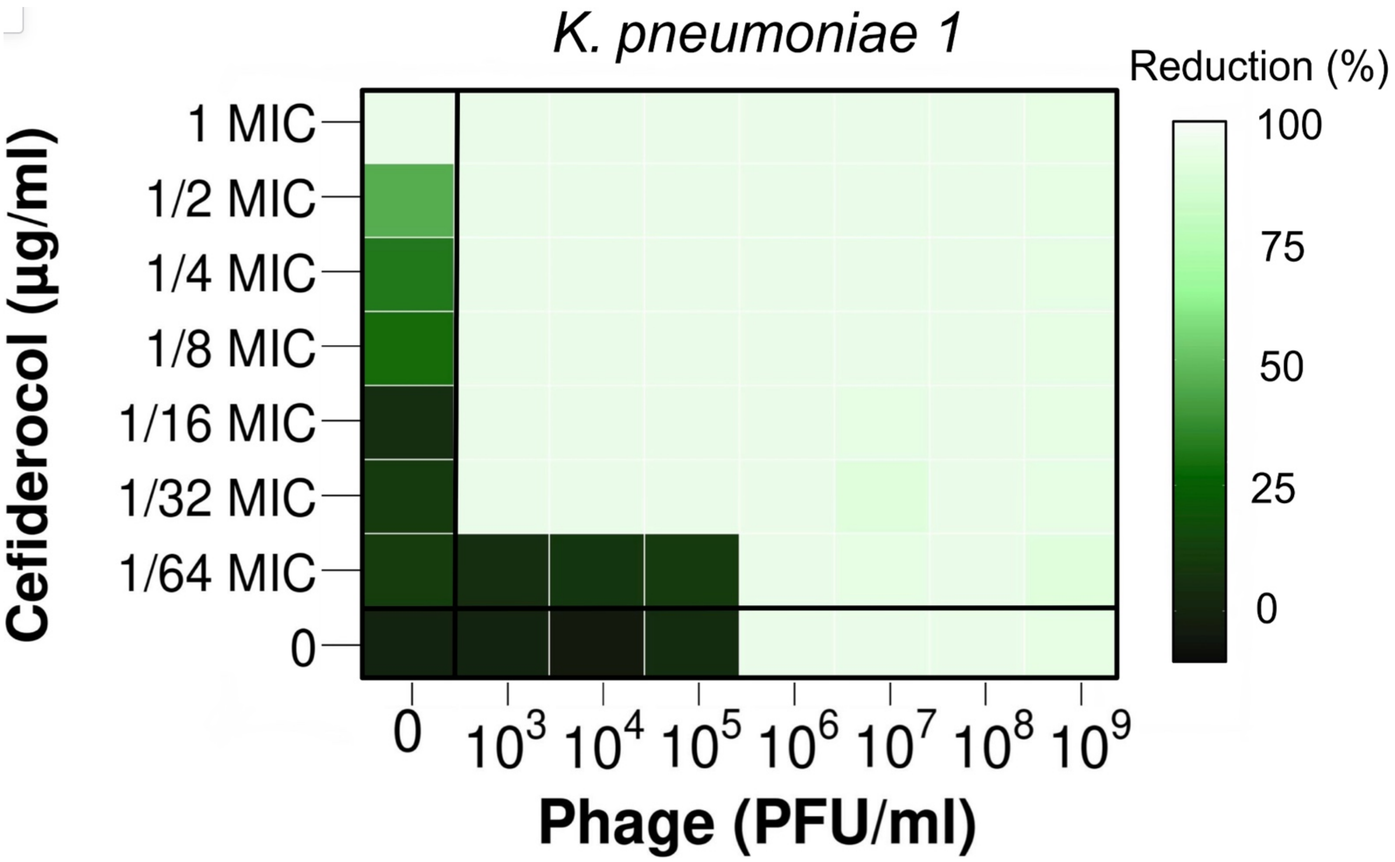

Reduction (%)

MIC	87	91	91	91	91	91	91	88
1/2 MIC	62	91	91	91	91	91	91	88
1/4 MIC	43	47	44	91	91	91	91	88
1/8 MIC	37	32	21	40	91	81	91	88
1/16 MIC	24	21	2	-5	-1	12	3	14
1/32 MIC	21	16	-6	-9	-12	-5	-7	13
1/64 MIC	5	11	6	9	9	-11	4	8
GC	0	15	12	15	10	11	7	5
		10Λ3	10Λ4	10Λ5	10Λ6	10Λ7	10Λ8	10Λ9

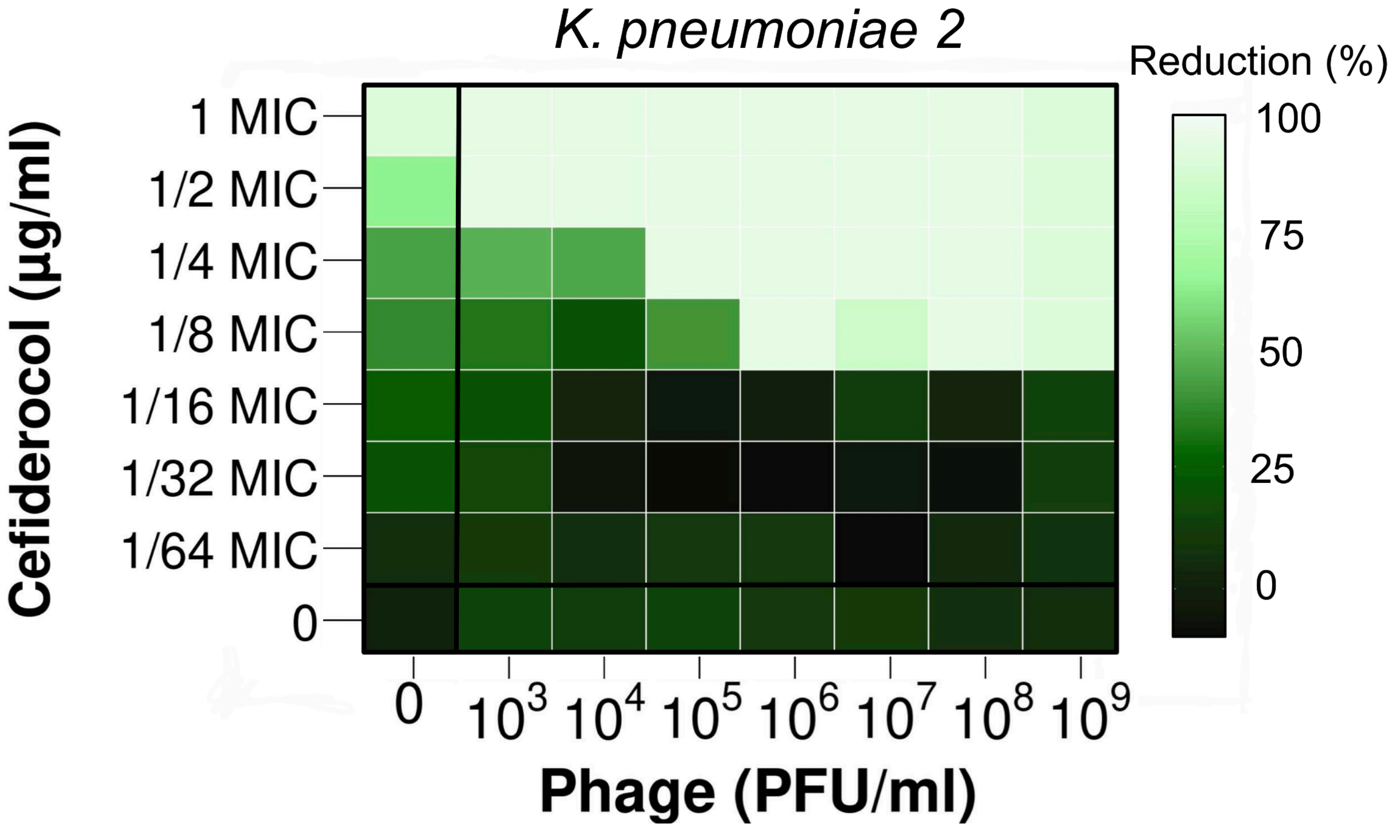

**Reduction** (%)								
MIC	93	94	94	94	94	93	93	93
1/2 MIC	50	94	92	94	93	94	94	93
1/4 MIC	44	94	94	94	93	94	94	93
1/8 MIC	29	93	94	94	94	94	94	93
1/16 MIC	35	94	94	94	94	94	94	93
1/32 MIC	19	26	94	94	94	94	94	93
1/64 MIC	19	19	34	15	32	59	94	93
GC	0	19	24	20	25	28	24	38
	GC	10Λ3	10Λ4	10Λ5	10Λ6	10Λ7	10Λ8	10Λ9

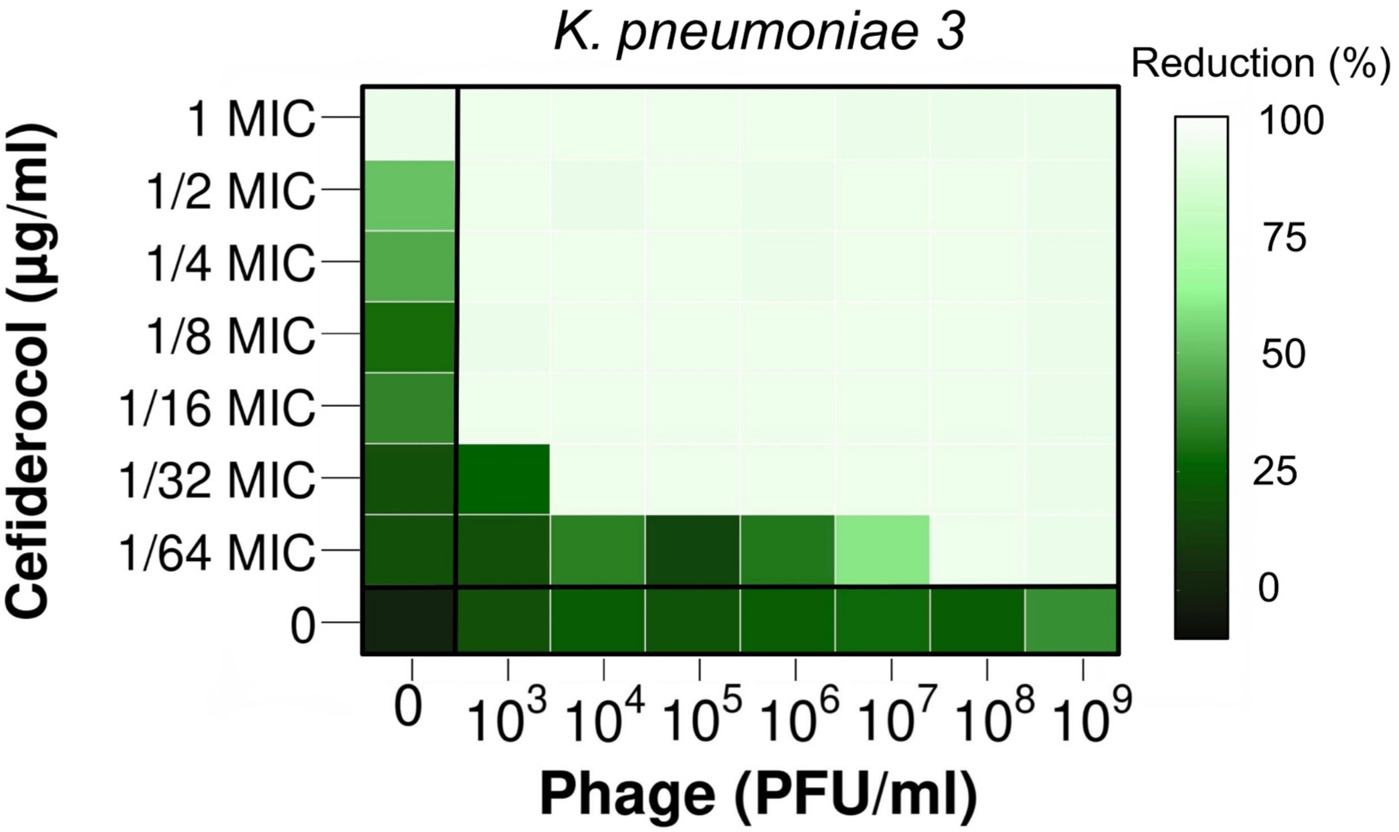

Reduction (%)								
MIC	96	95	96	95	96	96	96	96
1/2 MIC	78	96	96	95	96	95	92	95
1/4 MIC	75	95	96	96	96	96	96	94
1/8 MIC	68	95	96	95	96	96	96	94
1/16 MIC	13	93	94	94	94	94	94	95
1/32 MIC	6	89	89	89	88	85	86	86
1/64 MIC	0	86	84	78	78	75	80	83
GC	0	66	81	81	82	71	82	81
	GC	10Λ3	10Λ4	10Λ5	10Λ6	10Λ7	10Λ8	10Λ9

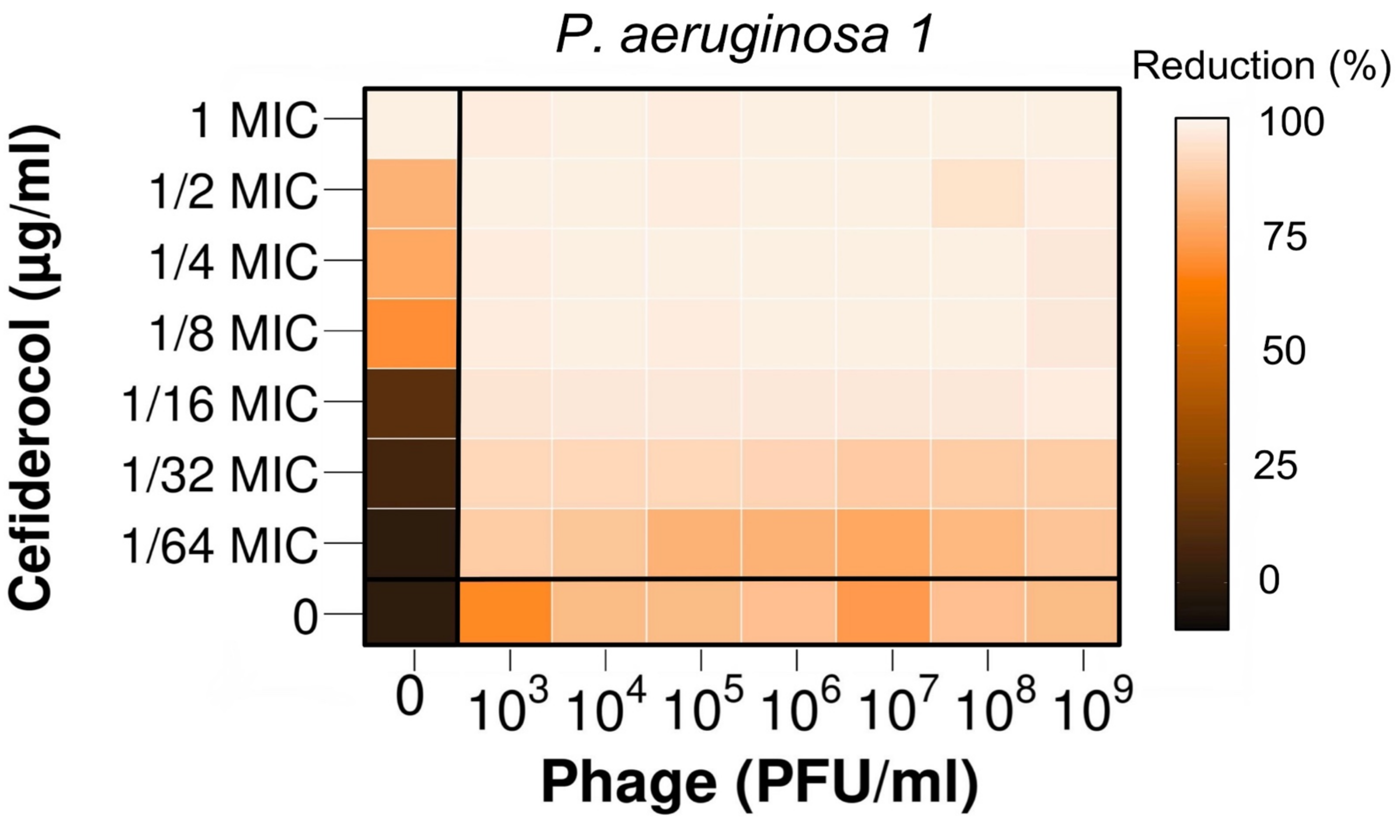

**Reduction** (%)								
MIC	95	95	95	95	95	95	95	95
1/2 MIC	83	95	95	95	95	95	95	95
1/4 MIC	31	95	95	95	95	95	95	95
1/8 MIC	24	94	95	95	95	95	95	95
1/16 MIC	11	95	95	95	95	95	95	94
1/32 MIC	1	95	94	95	95	95	95	95
1/64 MIC	0	95	95	95	95	95	95	95
GC	0	95	95	95	95	95	95	94
	GC	10^3	10^4	10^5	10^6	10^7	10^8	10^9

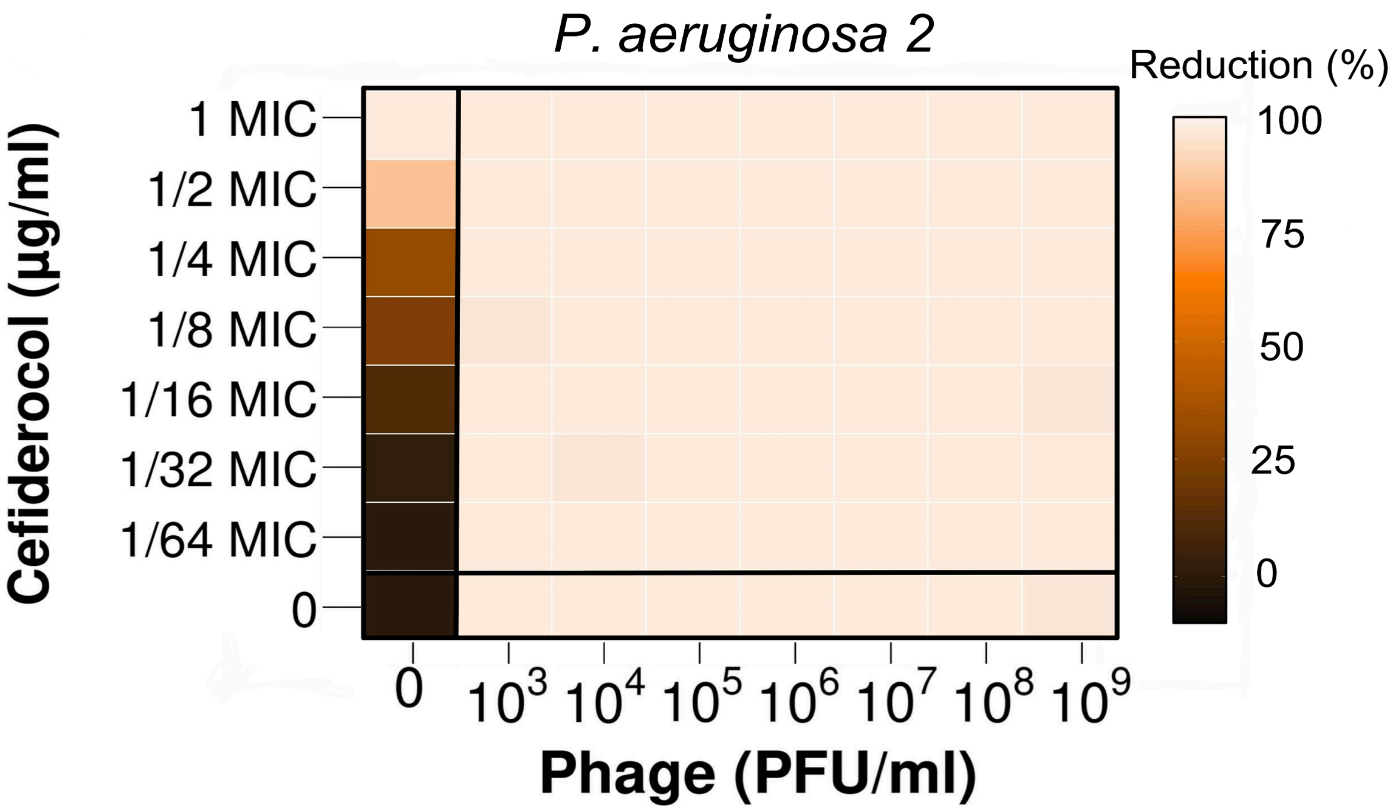

Reduction (%)								
MIC	96	96	96	96	96	96	96	96
1/2 MIC	19	79	80	87	95	96	96	95
1/4 MIC	13	38	52	45	71	95	96	96
1/8 MIC	9	13	40	34	46	84	96	96
1/16 MIC	13	10	36	28	38	64	88	96
1/32 MIC	26	15	18	31	22	36	70	86
1/64 MIC	31	18	7	6	14	21	48	73
GC	0	12	7	9	14	23	17	63
	GC	10∧3	10∧4	10∧5	10∧6	10∧7	10∧8	10∧9

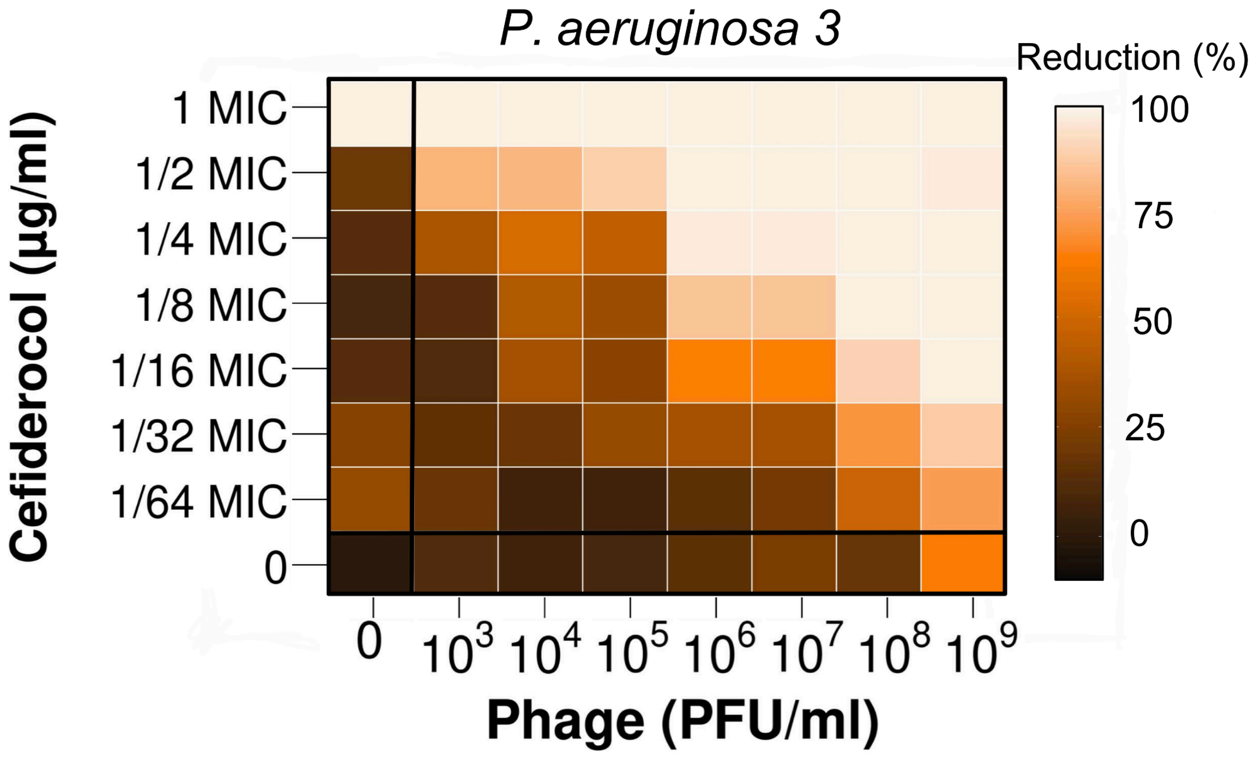

Supplement: Supplementary file 1 — Supplementary Material 1 [file 41598_2025_1704_MOESM1_ESM.pdf]
